# Gestational Age and Sex Influence the Susceptibility of Human Neural Progenitor Cells to Low Levels of MeHg

**DOI:** 10.1007/s12640-017-9786-x

**Published:** 2017-07-29

**Authors:** Karin Edoff, Marilena Raciti, Michaela Moors, Erik Sundström, Sandra Ceccatelli

**Affiliations:** 1 Department of Neuroscience, Karolinska Institutet, Retzius väg 8, SE-171 77 Stockholm, Sweden; 2 Department of Neurobiology, Care Sciences, and Society, Karolinska Institutet, Geriatrik-lab plan 5, SE-141 52 Huddinge, Sweden

**Keywords:** Developmental neurotoxicity, Methylmercury, Human neural progenitor cells, Cell migration, Sex-related differences

## Abstract

The developing nervous system is highly susceptible to methylmercury (MeHg), a widespread environmental neurotoxic contaminant. A wide range of morphological and functional outcomes have been described; however, there are still open questions regarding the mechanisms behind the developmental neurotoxic effects induced by low-level exposure. In the present study, we have examined the effects of nanomolar concentrations of MeHg on primary fetal human progenitor cells (hNPCs) with special focus on the role played by developmental stage and sex on the neurotoxic outcome. We found that neurospheres derived from earlier gestational time points exhibit higher susceptibility to MeHg, as they undergo apoptosis at a much lower dose (25 nM) as compared to neurospheres established from older fetuses (100 nM). At subapoptotic concentrations (10 nM), MeHg inhibited neuronal differentiation and maturation of hNPCs, as shown by a reduced number of Tuj1-positive cells and a visible reduction in neurite extension and cell migration, associated with a misregulation of Notch1 and BDNF signaling pathways. Interestingly, cells derived from male fetuses showed more severe alterations of neuronal morphology as compared to cells from females, indicating that the MeHg-induced impairment of neurite extension and cell migration is sex-dependent. Accordingly, the expression of the CDKL5 gene, a major factor regulating neurite outgrowth, was significantly more downregulated in male-derived cells. Altogether, gestational age and sex appear to be critical factors influencing in vitro hNPC sensitivity to low levels of MeHg.

**Electronic supplementary material:**

The online version of this article (doi:10.1007/s12640-017-9786-x) contains supplementary material, which is available to authorized users.

## Introduction

Methylmercury (MeHg) is a widespread environmental contaminant well known to be particularly harmful during nervous system development. The developmental neurotoxicity in humans has been recognized after poisoning catastrophes (Harada 1995), where children with neurological impairments were born by seemingly symptom-free women, highlighting that the developing nervous system is much more vulnerable than the adult one. Histopathological examinations during autopsies of MeHg exposed infants have shown alterations in specific brain areas, such as cerebellum and cerebral cortex (Roegge et al. 2006; Johansson et al. 2007), and signs of defects in neuronal organization and migration (Choi et al. 1978; Wilson et al. 2005; Fahrion et al. 2012).

Epidemiological data and behavioral studies on experimental animals exposed in utero have established that subcytotoxic doses that do not induce apoptosis or major histopathological signs still cause long-lasting impairments (Onishchenko et al. 2007; Johansson et al. 2007; Castoldi et al. 2008; Onishchenko et al. 2008). Moreover, behavioral studies in experimental animals, prenatally exposed to MeHg, showed in males but not in females reduced motor activity (Rossi et al. 1997; Giménez-Llort et al. 2001) and depression-like behavior (Onishchenko et al. 2007; Onishchenko et al. 2008).

The molecular mechanisms behind low-level MeHg-induced developmental neurotoxicity have been studied in different experimental models both in vivo and in vitro, and neural stem and progenitor cells have been shown to be among the most sensitive targets (Tamm et al. 2006; Johansson et al. 2007; Tamm et al. 2008). We have found that rat embryonic NPCs exposed to very low concentrations of MeHg undergo alterations in proliferation capacity and increased susceptibility to oxidative stress and that these changes are heritable as they are present in daughter cells never directly exposed to the neurotoxicant (Bose et al. 2012). These cellular alterations are accompanied by changes in DNA methylation, suggesting the involvement of epigenetic mechanisms (Bose et al. 2012). In addition, we found that male mice exposed to low levels of MeHg in utero exhibited reduced hippocampal neurogenesis even as adults and had fewer granule neurons in the dentate gyrus (Onishchenko et al. 2008).

To further investigate the mechanisms involved in low doses of MeHg neurotoxicity, we examined putative sex-related differences in the susceptibility to MeHg in primary human progenitor cells (hNPCs), with special focus on neuronal differentiation and maturation. Our data show that 10 nM MeHg inhibits neuronal differentiation and that the underlying mechanism probably targets the Notch signaling, a key regulator of neurogenesis (Louvi and Artavanis-Tsakonas 2006; Imayoshi et al. 2010).

There are several studies showing that MeHg disrupts neuronal migration (Heidemann et al. 2001; Moors et al. 2007; Moors et al. 2009; Guo et al. 2013), which may represent one of the main factors mediating MeHg developmental neurotoxicity. However, the mechanisms involved need to be further elucidated. Here we show that subapoptotic concentrations of MeHg induce an impaired migration associated to misexpression of cyclin-dependent kinase-like 5 (*CDKL5*), a key gene regulating neuronal morphogenesis and dendritic arborization by a mechanisms involving BDNF-Rac1 signaling (Chen et al. 2010). Moreover, MeHg interferes with neuronal maturation in a sex-dependent manner, as the observed alterations are more pronounced in cultures established from male fetuses.

## Material and Methods

### Chemicals

All chemicals were and reagents were obtained from Life Technologies and Sigma-Aldrich unless otherwise stated. MeHg hydroxide was purchased from ALFA, Johnson Matthey, Karlsruhe, Germany.

### Cell Culture

Postconception week (PCW) 8.5 hNPC cultures were established from human fetal central nervous system tissue. The Regional Ethics Committee, Stockholm, Sweden (nos. 2008/158-33/3, 2011/1101-32) approved the procedure. Briefly, cortical forebrain tissue was collected from clinical first trimester routine abortions, after obtaining informed consent by women undergoing termination of pregnancy. The human tissue was homogenized with a glass-Teflon homogenizer and cultured at 100,000–200,000 cells/ml in NS medium supplemented with 20 ng/ml epidermal growth factor (EGF), 20 ng/ml bFGF, and 10 ng/ml ciliary neurotrophic factor (CNTF) (all from R&D), as previously described (Åkesson et al. 2009). Neurospheres cultures were passaged every 7–14 days by using TrypLE Express (Life Technologies), and fresh medium was added twice a week. hNPC was expanded as free-floating neurospheres in Corning® non-treated culture dishes or Corning® ultra-low attachment culture dishes (100 mm × 20 mm, Sigma-Aldrich) and maintained in a humidified atmosphere at 37 °C and 5% CO_2_. All the following experiments were performed in hNPCs between passages 5 and 10.

hNPCs from PCW 16 were obtained from Lonza (Verviers SPRL) and cultured as previously described (Moors et al. 2009; Moors et al. 2012) at 37 °C and 5% CO_2_ as a suspension culture in defined serum-free media composed of Dulbecco’s modified Eagle medium (DMEM) and Hams F12 (3:1), supplemented with penicillin/streptomycin (50 U/ml), B27 1:50 (Invitrogen), 20 ng/ml EGF and 20 ng/ml recombinant human fibroblast growth factor (FGF; R&D Systems). Passaging was performed mechanically by cutting large spheres into smaller pieces using a McIlwain tissue chopper (Svendsen et al. 1998). Growing the human neural progenitor cells as neurospheres allows large numbers of cells to be expanded in small volumes of medium. However, to achieve homogenous levels of MeHg exposure for proliferation or differentiation assays, we dissociated the neurospheres to single cells before exposing them to MeHg containing medium. For migration assays, we used intact neurospheres.

All the experiments were performed using doses and times of exposure that do not induce apoptosis. For proliferation analyses, single cells were plated onto poly-d-lysine (PDL) and laminin coated glass coverslips (diameter 12 mm, placed in Nunclon® Δ Multidishes, 24 wells, flat bottom) and kept in DMEM/F12/ N2 (DFN) medium (DMEM/Hams F12 3:1, supplemented with N2, 1:100,Invitrogen), supplemented with FGF and EGF. The next day, cells were exposed to 10–100 nM MeHg in FGF/EGF-supplemented DFN medium for 24 h (see also supplementary material). The exposure was performed by replacing the culture medium with FGF/EGF-supplemented DFN medium containing MeHg. In the control cell cultures, no MeHg was added to the replacement medium. For the proliferation studies, more than 5100 cells/nuclei per treatment (in total) were counted.

For differentiation analyses, dissociated cells were plated on PDL and laminin coated glass coverslips (placed in Nunclon® Δ Multidishes, 24 wells, flat bottom) at a density of 40.000 cells per 12 mm coverslip, in MeHg-containing (10 nM MeHg) or MeHg-free DFN medium. Gene expression analysis, immunostaining, neurite length quantification, and apoptosis assays were performed after 96 h of differentiation. For the apoptosis studies, more than 3.300 cells/nuclei per treatment (in total) were counted.

### Sex Determination

**Fig. 1:**
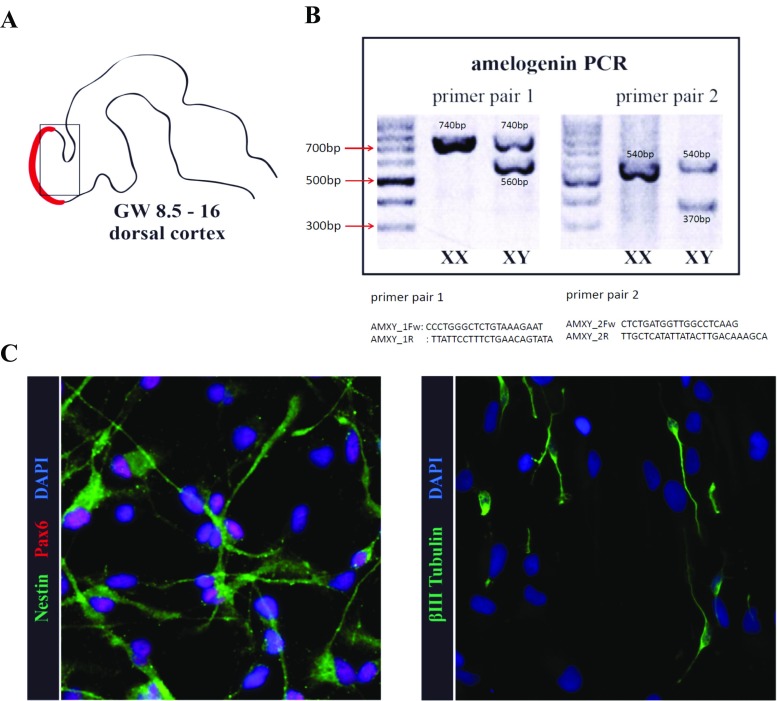
Human neural progenitor cell characterization. **a** Human NPCs were isolated from the dorsal cortex from terminated fetuses at PCW 8.5–16. **b** Cells were grown as neurospheres that can be expanded in vitro for multiple passages. Chromosomal *XX* or *XY* karyotypes of hNPCs were identified by PCR amplification of the amelogenin gene. **c** In the presence of EGF and FGF, cells showed typical morphology and gene expression pattern of radial glia. NESTIN is in *green*, PAX6 in *red*. **d** Eighteen hours after growth factor withdrawal, beta tubulin III (*green*)-positive neurons emerged. *Scale bars* represent 50 μM (**c**, **d**) (color figure online)

For sex determination, genomic DNA was harvested from about 50 neurospheres using a genomic DNA extraction kit. The DNA was eluted in water and subjected to PCR with two primer pairs against the *AMELX*/AMELY gene (Nakahori et al. 1991). *AMELX/AMELY* is a single copy gene, located on the X and Y chromosomes. X- and Y-specific products with different sizes were simultaneously detected because of difference in the lengths of corresponding introns (Fig. 1b).

### Immunohistochemistry, Fluorescence Microscopy, and Quantification

Cell cultures were fixed in 3% paraformaldehyde for 30 min at room temperature, then washed, and stored in phosphate-buffered saline (PBS). Apoptotic nuclei were detected by staining with Hoechst 33342, 1 μg/ml. Primary antibodies were rabbit anti-Ki67 (1:1000 Novcastra), β(III)tubulin (1:500, Covance), and rabbit-antiglial fibrillary acidic protein (GFAP; 1:500, DAKO).

Images were collected from random fields by a Nikon inverted fluorescent microscope (Nikon Eclipse Ti-S) equipped with a Nikon Digital Sight DS-Qi1MC camera. For quantitative analysis, the images were batch processed (to avoid bias) using the Volocity image analysis software (Demo-version, PerkinElmer) or ImageJ (http://imagej.net/ImageJ).

### Migration Assay

For migration assay, neurospheres were plated in PDL and laminin-coated multi-well plates (24 or 48 wells, Nunc) and left to attach overnight in DFN medium. The following morning, cultures were switched to MeHg-containing DFN medium (10 nM MeHg) or fresh MeHg-free DFN medium and placed in a Cell-IQ incubator (Chip-Man Technologies) for live imaging over the next 26 h. Phase contrast images of each neurospheres were collected every 30 min.

### RNA Extraction, cDNA Synthesis, and Quantitative RT-PCR

For mRNA extraction and quantification, total RNA was isolated using the peq Gold Microspin Total RNA Kit (peqLab GmbH, Erlangen, Germany). Complementary DNA (cDNA) was synthetized from at least 1 μg RNA by using Superscript II First-Strand cDNA Synthesis Kit according to the manufacturer’s protocol. Amplification reactions were set up, and product accumulation was measured by quantitative real-time (qRT) PCR analyses based on SYBR Green detection via ABI Prism 7000. Sequence Detection System with SDS software (version 2.1; Applied Biosystems, Foster City, CA). The qRT-PCR cycle conditions were 50 °C for 2 min, 95 °C for 10 min, 95 °C for 15 s, and 60 °C for 1 min (40 cycles). Expression levels were normalized to the housekeeping genes β-actin and ribosomal protein-like 13 (ΔCt = Ct (target gene) − Ct (housekeeping gene), which showed no MeHg-induced changes in gene expression (data not shown). Relative expression levels were calculated as ΔΔCt = ΔCtMeHg − ΔCtcontrol, and expression changes were calculated as 2−ΔΔCt. Primers were used at a final concentration of 4 μM. Primer sequences and annealing temperatures used for qRT-PCRs were as follows:

- HES5_fw 5′-ACATCCTGGAGATGGCTGTC-3′
- HES5_rev 5′-AGCAGCTTCATCTGCGTGT-3′, Ta = 58 °C
- BDNF_fw 5′-CAGTTGCGCGTTCTGAAATA-3′
- BDNF_rev 5′-CAGGGCTCTACCTTTTGCTT-3′, Ta = 58 °C
- CDKL5_fw 5′-ATCCAAAACCGTCTGAAGGA-3′
- CDKL5_rev 5′-CCTGCTAGAAGTGGGGGACT-3′, Ta = 58 °C
- AMXY_1_fw 5′-CCCTGGGCTCTGTAAAGAAT-3′
- AMXY_1_rev 5′-TTATTCCTTTCTGAACAGTATA-3′, Ta = 54 °C
- AMXY_2_fw 5′-CTCTGATGGTTGGCCTCAGG-3′
- AMXY_2_rev 5′-TTGCTCATATTATACTTGACAAAGCA-3′, Ta = 58 °C.

Product specificity was determined via melting curve analyses (temperature ramp from 60 to 95 °C) and agarose gel electrophoresis. All experiments were done on three replicate samples from two independent cell preparations from different donors.

### Statistics

All experiments were performed on cells from at least three different fetuses in at least two replicate cultures (see Table 1). For statistical analysis, ANOVA followed by Tukey’s post hoc test was used for comparisons between control and cultures exposed to different concentrations of MeHg. Factorial (two-way) ANOVA was used to relate MeHg exposure and sex differences. Student’s *t* test was used for comparisons of two groups. The significance value was set at *p* < 0.05. Values are shown as mean ± SEM unless otherwise stated.

**Table 1:** Number of fetuses employed for each type of experiment

Marker/assay	Fetuses	XY	XX
Ki67 staining	6	3	3
Tuji1 staining	8	4	4
GFAP staining	3	2	1
Neurite length assay	8	4	4
Hes5 qRT-PCR	8	4	4
BDNF qRT-PCR	8	4	4
CDKL5 qRT-PCR	8	4	4
Migration assay	8	4	4

## Results

### Fetus Developmental Age Influences Cytotoxicity of MeHg

The developing nervous system is particularly sensitive to insults during the first trimester of pregnancy (Miodovnik 2011). Therefore, as a first aim of the present study, we wanted to evaluate neurodevelopmental effects of low doses of MeHg in human neurospheres derived from PCW 8.5 fetuses (four male and four female), representative of an early stage of neurogenesis (Stiles and Jernigan 2010) (Fig. 1a).

Neurospheres generated from 8.5-week-old fetuses are characterized by the expression of well-established radial glia associated markers, namely Sox2, Pax6, and Nestin (Lendahl et al. 1990; Götz et al. 1998; Graham et al. 2003) (Fig. 1c). Clonal neurospheres are composed by heterogeneous cellular populations including neural stem cells and neuronal and glial progenitors in different stages of differentiation (Suslov et al. 2002; Jensen and Parmar 2006). Therefore, when dissociated neurospheres are differentiated, the progenitor population will rapidly initiate neuronal and glial differentiation (Fig. 1d), while the neural stem cell population will keep a radial glia-like morphology (Fig. 1c; supplementary Movie 1).

**Fig. 2:**
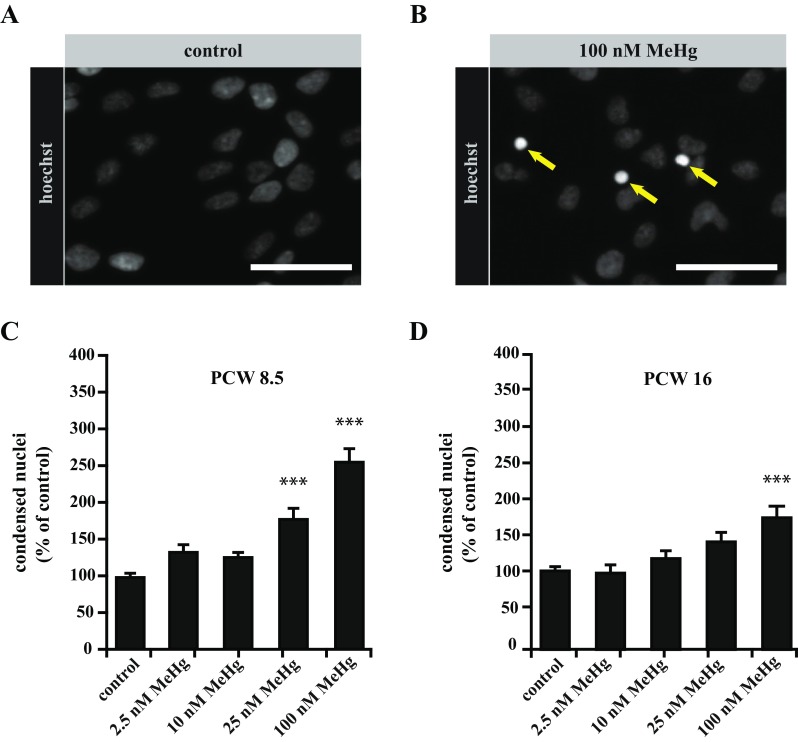
Assessment of hNPC susceptibility to MeHg at different gestational time points. **a**, **b** Apoptotic index in control and MeHg-treated cultures was evaluated after 4 days of differentiation using Hoechst 33342 to count nuclei with normal morphology and nuclei exhibiting apoptotic chromatin condensation. **c** In PCW 8.5 cultures, significantly increased levels of apoptosis were found after exposure to 25 nM and 100 nM MeHg. Control cultures exhibited 3.4% apoptosis (177 out of 5273 counted nuclei). **d** In PCW 16 cultures, only 100 nM MeHg increased apoptosis. Control cultures exhibited 5.4% apoptosis (305 out of 5682 counted nuclei). *Scale bars* represent 50 μM (**a**, **b**). *Error bars* represent SEM, ***p ≤ 0.001 (**c**, **d**)

To assess whether MeHg toxicity is influenced by the developmental stage, we used hNPC cultures also from PCW 16 fetuses and assayed the effects of four MeHg concentrations (in the range 2.5–100 nM) on the apoptosis rate. To this purpose, we evaluated chromatin condensation and quantified nuclei with apoptotic morphology (Darzynkiewicz et al. 1992) (Fig. 2a, b).

In hNPC cultures from PCW 8.5-week fetuses, we found significantly increased apoptosis after exposure to 25 nM MeHg (Fig. 2c), while cultures established from 16 weeks fetuses (PCW 16) showed a comparable amount of apoptotic cells only after exposure to 100 nM MeHg (Fig. 2d), indicating that the susceptibility of hNPCs to MeHg is related to the developmental stage.

### Subapoptotic Doses of MeHg Affect Neuronal Differentiation of PCW 8.5 hNPCs

**Fig. 3:**
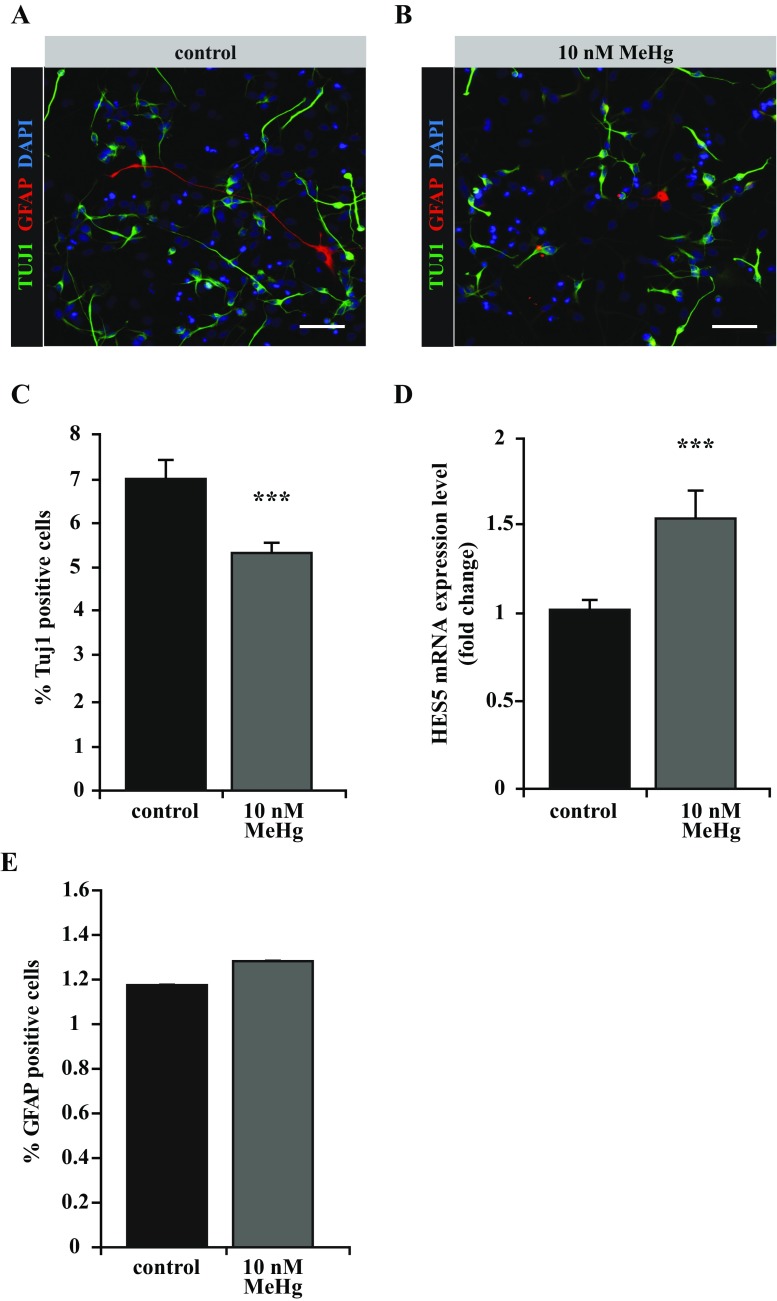
MeHg-induced impairment of hNPC neuronal differentiation. **a**, **b** Immunohistochemical stainings showing newly formed beta tubulin III (Tuj1)-positive neurons (*green*) and GFAP-positive astrocytes (*red*) in control and 10 nM MeHg-treated cultures, after 4 days of spontaneous differentiation. **c** Quantification of Tuj1-positive cells expressed as percentage of total cell numbers. **d** qRT-PCR quantification of HES5 expression level in control and MeHg treated cells. **e** Percentage of control and MeHg-treated cells immunoreactive for GFAP after 4 days of differentiation. *Scale bars* represent 50 μM (**a**, **b**). Error bars represent SEM, *** *p* ≤ 0.001 (**c**, **d**)

Next, we wanted to evaluate the effect on neuronal differentiation of 10 nM MeHg, a subtoxic concentration that does not affect the proliferation of PCW 8.5-derived hNPCs (see Suppl. Fig. 1). After 4 days of spontaneous differentiation, MeHg-treated hNPCs showed a significant reduction in the number of newly formed neurons (Tuj1-positive) (Fig. 3a, b, c). It is known that MeHg is able to activate the Notch signaling pathway by regulating ADAM metalloproteases (Bland and Rand 2006; Tamm et al. 2008); therefore, we looked at the expression of the well-known Notch signaling target HES5. Consistently, MeHg exposure induced an upregulation of HES5, as shown in Fig. 3d.

**Fig. 4:**
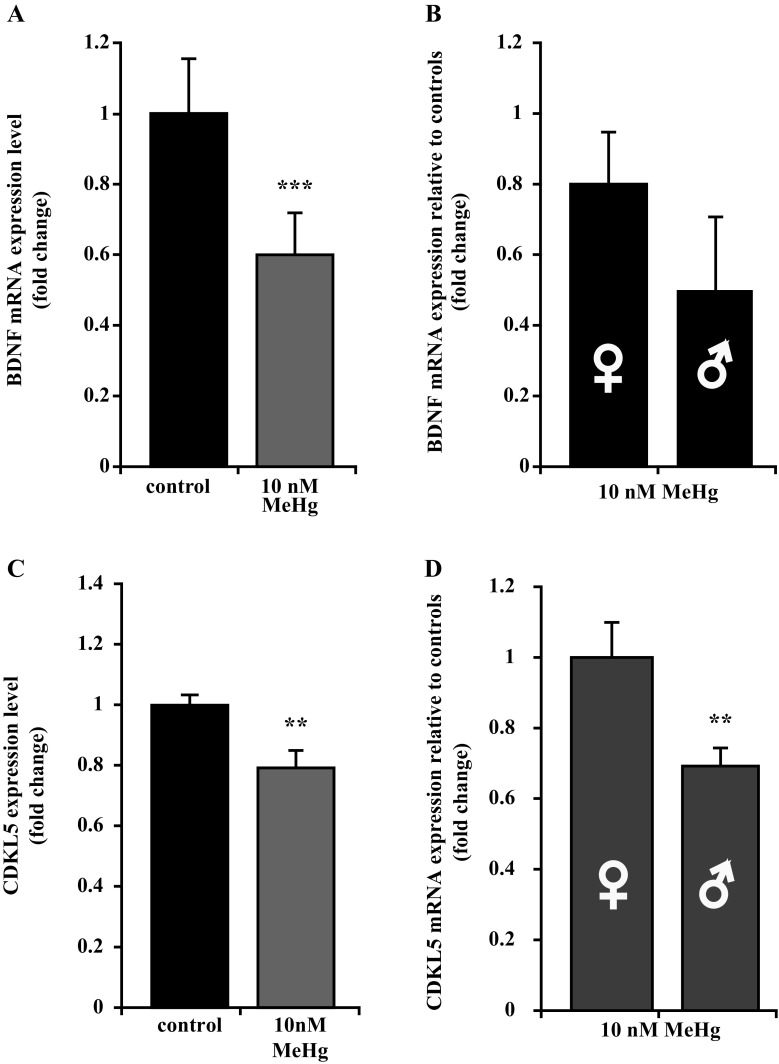
Effects of MeHg on BDNF and CDKL5 expression. **a**, **b** qRT-PCR quantification revealed BDNF gene downregulation following 10 nM MeHg exposure. No significant difference in BDNF expression level emerged by comparing male and female hNPC. **c** qRT-PCR experiment showing CDKL5 gene downregulation after 10 nM MeHg exposure. **d** The expression level of CDKL5 gene is affected differently in male versus female hNPCs after 10 nM MeHg treatment. *Error bars* represent SEM, *p < 0.05;**p ≤ 0.001 (**a**, **b**)

We next looked at the glial compartment and found no changes in the number of GFAP expressing cells (Fig. 3a, b, e), suggesting that MeHg stalls neuronal differentiation rather than causing a shift toward the glial fate. To assess whether the observed MeHg-induced inhibition of neuronal differentiation was sex-related, we compared the expression level of the same neuronal and glial markers in hNPCs from male and female fetuses in the presence of MeHg but we could not find any significant change (data not shown). Due to its crucial role in regulating neuronal differentiation (Numakawa et al. 2010), we quantified BDNF expression on differentiating PCW 8.5 hNPCs and found it to be significantly lower in cultures exposed to MeHg (Fig. 4a). There was no sex-related change in BDNF expression when comparing MeHg-treated male cells to female cells (Fig. 4b).

### MeHg Exposure Affects Neurite Extension and Neuronal Migration in a Sex-Related Manner

**Fig. 5:**
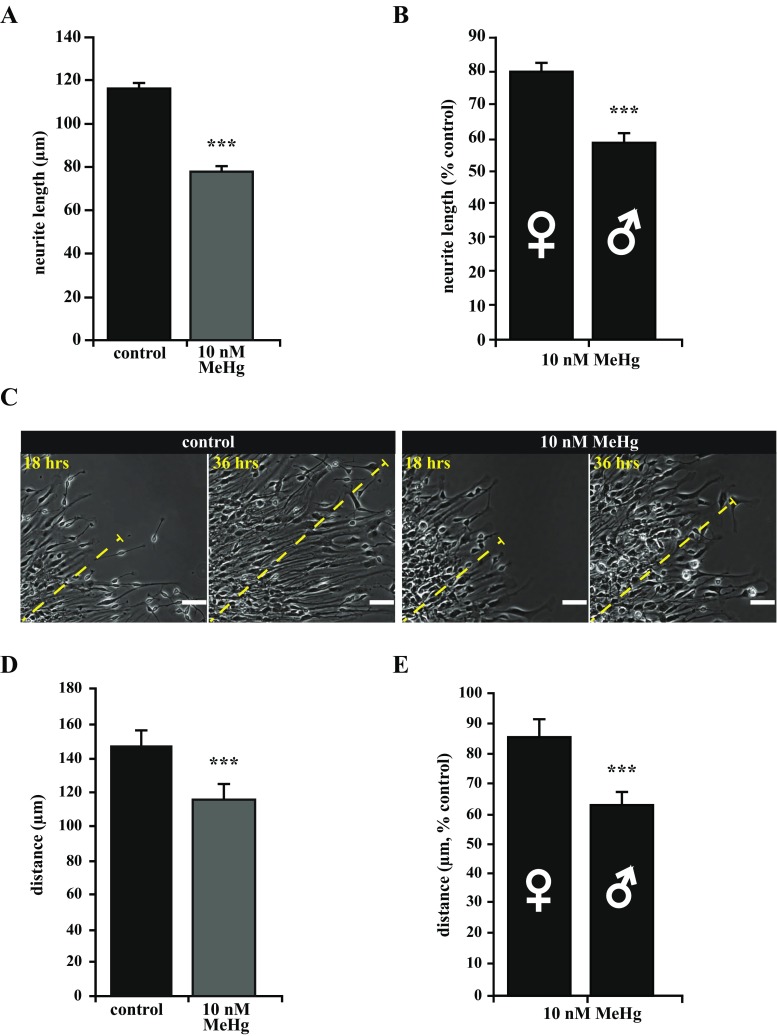
MeHg-induced disruption of neurite extension and cell migration. **a** Quantification of neurite length showed a significant difference in cultures treated with 10 nM MeHg compared to controls. **b** Moreover, neurites in male MeHg-treated cultures were significantly shorter than neurites of MeHg-treated female cultures. **c**, **d** In vivo, radial glia progenitors extend long processes to the pial surface. The extended processes serve as a scaffold for migration of newly generated neurons and direct cortical neurons to their final location. In vitro, human neural progenitors attached to a laminin-coated substrate extend processes that newly formed neurons migrate along. The distance of process extension/migration over 18 h was measured and quantified for control and 10 nM MeHg treated neurospheres. MeHg treatment caused a significant reduction in migration. **e** Relative to controls, there was a significant difference in the reduction of migration between female and male neurospheres. *Scale bars* represent 50 μM (**c**). *Error bars* represent SEM, ***p ≤ 0.001 (**a**, **b**; **d**, **e**)

We next measured the neurite length of immature neurons in cultures treated with 10 nM MeHg and found a significant difference after 4 days of spontaneous differentiation, as compared to control (Fig. 5a). Importantly, when comparing male versus female cultures, neurite length was significantly reduced in cultures of developing neurons with male karyotype (80% of control in females and 59% of control in males; Fig. 5b).

Moreover, MeHg exposure reduced cell migration over a period of 18 h to about 77% of control (Fig. 5c, d), and the male-karyotype cultures were more affected as compared to cultures from female karyotype (86% of control in females and 63% of control in males; Fig. 5e). In light of the CDKL5 (cyclin-dependent kinase-like 5) role as a critical regulator of neuronal morphogenesis (Chen et al. 2010), we looked at its expression in MeHg-exposed and control cultures of differentiating PCW 8.5 hNPCs. CDKL5 expression was significantly decreased in MeHg-treated cultures, and the expression was even lower in cultures with male karyotype, as compared to female karyotype cells (Fig. 4c, d).

## Discussion

In the present study, we show that the cytotoxicity of low doses of MeHg in human neurospheres is influenced by gestational age and that subapoptotic concentrations of MeHg impair neuronal maturation in a sex-dependent manner.

We found that hNPCs from an earlier fetal period are more susceptible to low doses MeHg as compared to cells from older fetuses, suggesting that MeHg toxicity is influenced by fetal developmental age and it is tempting to speculate that the molecular mechanisms involved may be related to the antioxidative enzymes expression level.

Previous studies from our group have shown that prenatal exposure to MeHg in the nanomolar range can inhibit neuronal differentiation (Tamm et al. 2008; Bose et al. 2012). In close accordance, the present data indicate that exposure to subapoptotic concentrations of MeHg interferes with neuronal differentiation of hNPCs, as shown by a decreased number of Tuj1-positive cells. It is well established that Notch signaling plays a key role in neurogenesis as its activation induces the expression of transcriptional repressor genes, including *Hes1* and *Hes5*, leading to the inhibition of neuronal differentiation (Imayoshi et al. 2010). In light of its role as Notch effector, the increased expression of HES5 observed in our cells exposed to MeHg suggests that the molecular mechanism underlying the decreased neuronal differentiation involves an over-activation of Notch signaling that keeps progenitor cells in their undifferentiated state. This is further supported by our data showing that the inhibition of neuronal differentiation is not associated with an increase in the number of glial cells, indicating that MeHg does not induce a shift toward the glial fate and non-neuronal cells retain their progenitor identity as a consequence of Notch signaling activation.

In the attempt to identify additional factors mediating MeHg detrimental effects on hNPC, we analyzed BDNF expression, a key factor for neuronal differentiation and survival (Numakawa et al. 2010). According to previous studies, perinatal exposure to MeHg induces an increase in DNA methylation and a concomitant decrease of H3 acetylation in the BDNF promoter region, leading to the repression of its expression (Tsankova et al. 2006; Onishchenko et al. 2008). Consistently, we found a decreased BDNF expression level in MeHg-exposed hNPC, suggesting that alterations in this pathway may be directly involved in the disruption of hNPC differentiation following MeHg treatment.

After a deeper characterization of differentiated hNPC, we found a reduced neurite extension and cell migration following exposure to MeHg. As previously shown, exposure to nM concentration of MeHg disrupts neuronal migration and inhibits axonal morphogenesis of NPCs in vivo and in vitro (Heidemann et al. 2001; Moors et al. 2007; Moors et al. 2009; Guo et al. 2013); however, the underlying mechanisms are still not clearly identified. A study by Guo et al. showed that exposure to low levels of MeHg suppresses the expression of three key proteins involved in the regulation of neuronal migration, namely Rac1, Cdc42, and RhoA (Guo et al. 2013). Another reasonable molecular mechanism may involve the BDNF-activated pathways. Indeed, it is known that after BDNF binding to TrkB receptor, several signaling pathways are activated, including the MAPK/ERK1/2, a crucial pathway promoting cell migration (Huang et al. 2004; Moors et al. 2007). Thus, the BDNF downregulation observed in our samples after MeHg exposure may contribute to the migration impairment.

Neuronal process elongation and maintenance are regulated by nerve growth factor (NGF) in a microtubule-dependent manner (Drubin et al. 1985), and it is known that MeHg interferes with the axonal outgrowth process by poisoning microtubule assembly (Miura et al. 2000; Heidemann et al. 2001). A recent study by Fujimura et al. (2016) showed that prenatal exposure to low-dose MeHg was associated with a significant downregulation of eukaryote elongation factor 1A1 (eEF1A1), a key factor regulating neurite outgrowth, through NGF/TrkA activated pathway (Fujimura et al. 2016). According to other studies (Inamura et al. 2005), additional mechanisms involving BDNF-mediated regulation of eEF1A activity play a role in the MeHg-induced inhibition of neurite extension. Indeed, it has been shown that BDNF promotes eEF1A phosphorylation and that the consequent increased eEF1A activity leads to an enhanced protein synthesis, resulting in the promotion of neurite extension in cortical neurons (Inamura et al. 2005). Therefore, it is likely that the BDNF downregulation observed in MeHg-exposed cells results in a further decrease of EF1A activity, which may crucially contribute to the detrimental effects on neurites extension. However, more experiment is needed to further support this hyphotesis.

It is worth noting that in our study, MeHg induced a reduction in the expression level of CDKL5, a critical gene regulating neuronal morphogenesis which is mutated in the Hanefeld variant of Rett syndrome (Chen et al. 2010). CDKL5 has been shown to exert different effects within neuronal cells depending on its subcellular location, i.e., cytoplasmic or nuclear (Rusconi et al. 2008). In the cytoplasm, CDKL5 regulates neuronal morphogenesis and dendritic arborization by a mechanism involving BDNF-Rac1 signaling (Chen et al. 2010). Indeed, it has been suggested that BDNF activates CDKL5 that, in turn, triggers Rac1 activity to regulate neuronal morphogenesis through the actin cytoskeleton remodeling (Chen et al. 2010). Thus, it is likely that the downregulation of CDKL5 observed in our MeHg-exposed cells prevents the BDNF-mediated activation of Rac1, leading to the defective axonal morphogenesis.

An interesting phenomenon observed in this study is the sex dependence of the MeHg-induced defects in neurite extension and cell migration, which were more pronounced in cultures established from male fetuses. This is in agreement with epidemiological and experimental studies showing that males are more susceptible to MeHg neurotoxicity as compared to females (McKeown-Eyssen et al. 1983; Rossi et al. 1997; Grandjean et al. 1998; Giménez-Llort et al. 2001; Björklund et al. 2007). As reported in previous studies, there are sex-related differences in the antioxidant defense system activity and in the peroxide production (Carrillo et al. 1992; Borrás et al. 2003). Indeed, it has been shown that mitochondria from female rats produce less peroxide than those from male rats of the same age (Borrás et al. 2003). Moreover, both gene expression level and enzymatic activity of Mn-superoxide dismutase and glutathione peroxidase were found to be significantly higher in female as compared to male rats (Borrás et al. 2003). In this regard, it is important to underline that a major event mediating MeHg-induced neurotoxicity is represented by its interaction with thiols from GSH (Sumi 2008; Farina et al. 2010). The subsequent decreased capacity of the entire antioxidant GSH system (Shanker et al. 2005; Stringari et al. 2008) may likely lead to the increased susceptibility to MeHg observed in cells from male fetuses.

In conclusion, the present study shows that the gestational age is a critical factor influencing hNPC sensitivity to low levels of MeHg. Subcytotoxic doses of MeHg impair neuronal differentiation and maturation of hNPC in a sex-dependent manner as shown by the more pronounced inhibition of neurites outgrowth and cell migration in MeHg-exposed cells from male fetuses. Our data point to Notch, CDNK5, and BDNF as critical players in the cascade of intracellular events leading to MeHg-induced in vitro neurotoxicity.

## Electronic supplementary material

ESM 1 (DOCX 20 kb).

ESM 2 (JPEG 153 kb).

High resolution image (EPS 905 kb).

ESM 3 (WMV 5705 kb).

## References

[CR1] Akesson E, Wolmer-Solberg N, Cederarv M, Falci S, Odeberg J (2009). Human neural stem cells and astrocytes, but not neurons, suppress an allogeneic lymphocyte response. Stem Cell Res.

[CR2] Björklund O, Kahlström J, Salmi P, Ogren SO, Vahter M, Chen JF, Fredholm BB, Daré E (2007). The effects of methylmercury on motor activity are sex- and age-dependent, and modulated by genetic deletion of adenosine receptors and caffeine administration. Toxicology.

[CR3] Bland C, Rand MD (2006). Methylmercury induces activation of Notch signaling. Neurotoxicology.

[CR4] Borrás C, Sastre J, García-Sala D, Lloret A, Pallardó FV, Viña J (2003). Mitochondria from females exhibit higher antioxidant gene expression and lower oxidative damage than males. Free Radic Biol Med.

[CR5] Bose R, Onishchenko N, Edoff K, Janson Lang AM, Farina S (2012). Inherited effects of low-dose exposure to methylmercury in neural stem cells. Toxicol Sci.

[CR6] Carrillo MC, Kanai S, Sato Y, Kitani K (1992). Age-related changes in antioxidant enzyme activities are region and organ, as well as sex, selective in the rat. Mech Ageing Dev.

[CR7] Castoldi AF, Johansson C, Onishchenko N, Coccini T, Roda E, Vahter M, Ceccatelli S, Manzo L (2008). Human developmental neurotoxicity of methylmercury: impact of variables and risk modifiers. Regul Toxicol Pharmacol.

[CR8] Chen Q, Zhu YC, Yu J, Miao S, Zheng J, Xu L, Zhou Y, Li D, Zhang C, Tao J, Xiong ZQ (2010). CDKL5, a protein associated with rett syndrome, regulates neuronal morphogenesis via Rac1 signaling. J Neurosci.

[CR9] Choi BH, Lapham LW, Amin-Zaki L, Saleem T (1978). Abnormal neuronal migration, deranged cerebral cortical organization, and diffuse white matter astrocytosis of human fetal brain: a major effect of methylmercury poisoning in utero. J Neuropathol Exp Neurol.

[CR10] Darzynkiewicz Z, Bruno S, Del Bino G, Gorczyca W, Hotz MA, Lassota P, Traganos F (1992). Features of apoptotic cells measured by flow cytometry. Cytometry.

[CR11] Drubin DG, Feinstein SC, Shooter EM, Kirschner MW (1985) Nerve growth factor-induced neurite outgrowth in PC12 cells involves the coordinate induction of microtubule assembly and assembly-promoting factors. J Cell Biol. doi:10.1083/jcb.101.5.1799 10.1083/jcb.101.5.1799 PMC2113947 2997236

[CR12] Fahrion JK, Komuro Y, Li Y, Ohno N, Littner Y, Raoult E, Galas L, Vaudry D, Komuro H (2012). Rescue of neuronal migration deficits in a mouse model of fetal Minamata disease by increasing neuronal Ca2+ spike frequency. Proc Natl Acad Sci.

[CR13] Farina M, Rocha JBT, Aschner M (2010). Oxidative stress and methylmercury-induced neurotoxicity. Developmental neurotoxicology research.

[CR14] Fujimura M, Usuki F, Cheng J, Zhao W (2016). Prenatal low-dose methylmercury exposure impairs neurite outgrowth and synaptic protein expression and suppresses TrkA pathway activity and eEF1A1 expression in the rat cerebellum. Toxicol Appl Pharmacol.

[CR15] Giménez-Llort L, Ahlbom E, Daré E, Vahter M, Ögren S, Ceccatelli S (2001). Prenatal exposure to methylmercury changes dopamine-modulated motor activity during early ontogeny: age and gender-dependent effects. Environ Toxicol Pharmacol.

[CR16] Götz M, Stoykova A, Gruss P (1998). Pax6 controls radial glia differentiation in the cerebral cortex. Neuron.

[CR17] Graham V, Khudyakov J, Ellis P, Pevny L (2003). SOX2 functions to maintain neural progenitor identity. Neuron.

[CR18] Grandjean P, Weihe P, White RF, Debes F (1998). Cognitive performance of children prenatally exposed to “safe” levels of methylmercury. Environ Res.

[CR19] Guo BQ, Yan CH, Cai SZ, Yuan XB, Shen XM (2013). Low level prenatal exposure to methylmercury disrupts neuronal migration in the developing rat cerebral cortex. Toxicology.

[CR20] Harada M (1995). Minamata disease: methylmercury poisoning in Japan caused by environmental pollution. Crit Rev Toxicol.

[CR21] Heidemann SR, Lamoureux P, Atchison WD (2001). Inhibition of axonal morphogenesis by nonlethal, submicromolar concentrations of methylmercury. Toxicol Appl Pharmacol.

[CR22] Huang C, Jacobson K, Schaller MD (2004). MAP kinases and cell migration. J Cell Sci.

[CR23] Imayoshi I, Sakamoto M, Yamaguchi M, Mori K, Kageyama R (2010). Essential roles of Notch signaling in maintenance of neural stem cells in developing and adult brains. J Neurosci.

[CR24] Inamura N, Nawa H, Takei N (2005). Enhancement of translation elongation in neurons by brain-derived neurotrophic factor: implications for mammalian target of rapamycin signaling. J Neurochem.

[CR25] Jensen JB, Parmar M (2006). Strengths and limitations of the neurosphere culture system. Mol Neurobiol.

[CR26] Johansson C, Castoldi AF, Onishchenko N, Manzo L, Vahter M, Ceccatelli S (2007). Neurobehavioural and molecular changes induced by methylmercury exposure during development. Neurotox Res.

[CR27] Lendahl U, Zimmerman LB, McKay RD (1990). CNS stem cells express a new class of intermediate filament protein. Cell.

[CR28] Louvi A, Artavanis-Tsakonas S (2006). Notch signalling in vertebrate neural development. Nat Rev Neurosci.

[CR29] McKeown-Eyssen GE, Ruedy J, Neims A (1983). Methyl mercury exposure in northern Quebec. II. Neurologic findings in children. Am J Epidemiol.

[CR30] Miodovnik A (2011). Environmental neurotoxicants and developing brain. Mt Sinai J Med A J Transl Pers Med.

[CR31] Miura K, Himeno S, Koide N, Imura N (2000). Effects of methylmercury and inorganic mercury on the growth of nerve fibers in cultured chick dorsal root ganglia. Tohoku J Exp Med.

[CR32] Moors M, Cline JE, Abel J, Fritsche E (2007). ERK-dependent and -independent pathways trigger human neural progenitor cell migration. Toxicol Appl Pharmacol.

[CR33] Moors M, Rockel TD, Abel J, Cline JE, Gassmann K, Schreiber T, Schuwald J, Weinmann N, Fritsche E (2009). Human neurospheres as three-dimensional cellular systems for developmental neurotoxicity testing. Environ Health Perspect.

[CR34] Moors M, Bose R, Johansson-Haque K, Edoff K, Okret S, Ceccatelli S (2012). Dickkopf 1 mediates glucocorticoid-induced changes in human neural progenitor cell proliferation and differentiation. Toxicol Sci.

[CR35] Nakahori Y, Takenaka O, Nakagome Y (1991). A human X-Y homologous region encodes “amelogenin”. Genomics.

[CR36] Numakawa T, Suzuki S, Kumamaru E, Adachi N, Richards M, Kunugi H (2010). BDNF function and intracellular signaling in neurons. Histol Histopathol.

[CR37] Onishchenko N, Tamm C, Vahter M, Hökfelt T, Johnson JA, Johnson DA, Ceccatelli S (2007). Developmental exposure to methylmercury alters learning and induces depression-like behavior in male mice. Toxicol Sci.

[CR38] Onishchenko N, Karpova N, Sabri F, Castrén E, Ceccatelli S (2008). Long-lasting depression-like behavior and epigenetic changes of BDNF gene expression induced by perinatal exposure to methylmercury. J Neurochem.

[CR39] Roegge CS, Morris JR, Villareal S, Wang VC, Powers BE, Klintsova AY, Greenough WT, Pessah IN, Schantz SL (2006). Purkinje cell and cerebellar effects following developmental exposure to PCBs and/or MeHg. Neurotoxicol Teratol.

[CR40] Rossi AD, Ahlbom E, Ogren SO, Nicotera P, Ceccatelli S (1997). Prenatal exposure to methylmercury alters locomotor activity of male but not female rats. Exp Brain Res.

[CR41] Rusconi L, Salvatoni L, Giudici L, Bertani I, Kilstrup-Nielsen C, Broccoli V, Landsberger N (2008). CDKL5 expression is modulated during neuronal development and its subcellular distribution is tightly regulated by the C-terminal tail. J Biol Chem.

[CR42] Shanker G, Syversen T, Aschner JL, Aschner M (2005). Modulatory effect of glutathione status and antioxidants on methylmercury-induced free radical formation in primary cultures of cerebral astrocytes. Brain Res Mol Brain Res.

[CR43] Stiles J, Jernigan TL (2010). The basics of brain development. Neuropsychol Rev.

[CR44] Stringari J, Nunes AK, Franco JL, Bohrer D, Garcia SC, Dafre AL, Milatovic D, Souza DO, Rocha JB, Aschner M, Farina M (2008). Prenatal methylmercury exposure hampers glutathione antioxidant system ontogenesis and causes long-lasting oxidative stress in the mouse brain. Toxicol Appl Pharmacol.

[CR45] Sumi D (2008). Biological effects of and responses to exposure to electrophilic environmental chemicals. J Health Sci.

[CR46] Suslov ON, Kukekov VG, Ignatova TN, Steindler DA (2002). Neural stem cell heterogeneity demonstrated by molecular phenotyping of clonal neurospheres. Proc Natl Acad Sci U S A.

[CR47] Svendsen CN, ter Borg MG, Armstrong RJ, Rosser AE, Chandran S, Ostenfeld T, Caldwell MA (1998) A new method for the rapid and long term growth of human neural precursor cells. J Neurosci Methods 85(2):141–52. doi:10.1016/S0165-0270(98)00126-5 10.1016/s0165-0270(98)00126-5 9874150

[CR48] Tamm C, Duckworth J, Hermanson O, Ceccatelli S (2006). High susceptibility of neural stem cells to methylmercury toxicity: effects on cell survival and neuronal differentiation. J Neurochem.

[CR49] Tamm C, Duckworth JK, Hermanson O, Ceccatelli S (2008). Methylmercury inhibits differentiation of rat neural stem cells via Notch signalling. Neuroreport.

[CR50] Tsankova NM, Berton O, Renthal W, Kumar A, Neve RL, Nestler EJ (2006). Sustained hippocampal chromatin regulation in a mouse model of depression and antidepressant action. Nat Neurosci.

[CR51] Wilson DT, Polunas MA, Zhou R, Halladay AK, Lowndes HE, Reuhl KR (2005). Methylmercury alters Eph and Ephrin expression during neuronal differentiation of P19 embryonal carcinoma cells. Neurotoxicology.

