# Supplementary material for: Gestational Age and Sex Influence the Susceptibility of Human Neural Progenitor Cells to Low Levels of MeHg
Source: Neurotox Res. 2017 Jul 29;32(4):683–93. doi: 10.1007/s12640-017-9786-x (PMC5602033; doi:10.1007/s12640-017-9786-x)
Supplement: Supplementary file 1 — (DOCX 20 kb). [file 12640_2017_9786_MOESM1_ESM.docx]

*Journal:*

*Neurotoxicity Research*

*Title:*

*Gestational age and sex influence the susceptibility of human neural progenitor cells to low levels of MeHg*

*Authors:*

*Karin Edoff^1*^, Marilena Raciti^1*#^, Michaela Moors^1^, Erik Sundström^2^, Sandra Ceccatelli^1^*

*Affiliations:*

*^1^Karolinska Institutet, Department of Neuroscience, Retzius väg 8, 171 77, Stockholm, Sweden.*

*^2^Karolinska Institutet, Department of Neurobiology, Care Sciences and Society, Geriatrik-lab plan 5, 14152 Huddinge, Sweden.*

**Equally contributing authors*

*#Corresponding author*

*Marilena.Raciti@ki.se*

*Telephone: +46-(0)8-524 875 86*

*Fax: +46-8-333864*

*Effect of low doses MeHg on hNPCs proliferation rate* .

To assess the MeHg concentration able to affect proliferation capacity in PCW 8.5- derived hNPCs, we quantified the proliferation marker Ki67 in cell cultures exposed to different concentration of MeHg for 24 hours. Ki67 is a nuclear protein expressed during all phases of the cell cycle, but absent from resting cells, and extensively used to determine the growth fraction of a cell population.We found that in untreated cells, the 35% of the cells expressed Ki67 (Suppl. Fig.1A and C), with no significant karyotype-related differences (data not shown). Looking at MeHg- exposed samples, we noticed that the proliferative capability was significantly reduced in cultures exposed to100 nM MeHg for 24 hours (Suppl. Fig.1B and C), while MeHg subcytotoxic dose (10 nM) did not caused any significant changes in the number of Ki67-expressing cells as compared to untreated controls (Suppl. Fig.1C).

*Figure Caption*

**Supplementary Fig.1***. Effect of MeHg nanomolar concentrations on the proliferation rate*. (A-B) Immunohistochemical stainings showing Ki67-positive cells (red) in relation to total cell numbers (DAPI in blue) in control and MeHg treated cultures. (C) Ki67 is a nuclear protein expressed during all active phases of the cell extensively used to determine the growth fraction of a cell population (Scholzen and Gerdes 2000). Quantification showed that in cultures established from fetuses at PCPCW 8.5 an average of 35% of the cells in control cultures expressed Ki67. In cultures exposed to 100 nM MeHg for 24 hrs there was a significant change in the number of Ki67-expressing cells compared to controls, while the subcytotoxic dose 10 nM MeHg didn’t affect the proliferation rate. Scale bars represent 50 µM (A-B). Error bars represent SEM, ***p≤ 0.001 (C)

References

Scholzen T, Gerdes J (2000) The Ki-67 protein: from the known and the unknown. J Cell Physiol 182:311–22. doi: 10.1002/(SICI)1097-4652(200003)182:3<311::AID-JCP1>3.0.CO;2-9
