# Supplementary figures and images for: Gestational Age and Sex Influence the Susceptibility of Human Neural Progenitor Cells to Low Levels of MeHg
Source: Neurotox Res. 2017 Jul 29;32(4):683–93. doi: 10.1007/s12640-017-9786-x (PMC5602033; doi:10.1007/s12640-017-9786-x)

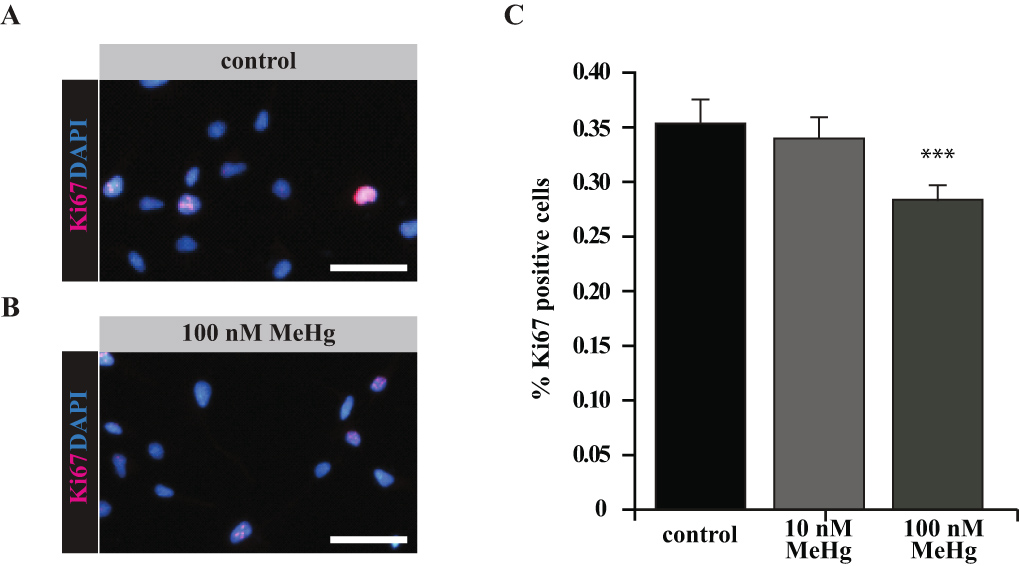

Supplement: Supplementary file 2 — (JPEG 153 kb). [file 12640_2017_9786_Fig6_ESM.jpg]
